# First-principles studies of defect behaviour in bismuth germanate

**DOI:** 10.1038/s41598-022-18586-x

**Published:** 2022-09-21

**Authors:** Salawu Omotayo Akande, Othmane Bouhali

**Affiliations:** Texas A & M University at Qatar, PO Box 23874 Doha, Qatar

**Keywords:** Chemistry, Materials science, Physics

## Abstract

Intrinsic defects are known to greatly affect the structural and electronic properties of scintillators thereby impacting performance when these materials are in operation. In order to overcome this effect, an understanding of the defect process is required for the design of more stable materials. Here we employed density functional theory calculations and the PBE0 hybrid functional to study the structural, electronic,defect process and optical properties of $\hbox {Bi}_4\hbox {Ge}_3\hbox {O}_{{12}}$ (BGO), a well know material used as scintillator. We examined possible intrinsic defects and calculated their formation energy and their impact on the properties that affect the scintillation process. Furthermore, we investigated the effect and role of rare earth element (REE = Nd, Pr, Ce and Tm) doping on the properties of the BGO system. While the PBE functional underestimated the band gap, the PBE0 was found to adequately describe the electronic properties of the system. Out of all the defects types considered, it was found that $\hbox {Bi}_{{Ge}}$ antisite is the most favourable defect. Analysis of the effect of this defect on the electronic properties of BGO revealed an opening of ingap states within the valence band. This observation suggests that the $\hbox {Bi}^{3+}$ could be a charge trapping defect in BGO. We found that the calculated dopant substitution formation energy increases with increase in the size of the dopant and it turns out that the formation of O vacancy is easier in doped systems irrespective of the size of the dopant. We analyzed the optical spectra and noted variations in different regions of the photon energy spectra.

## Introduction

Scintillators are materials that convert high energy rays such as X-rays and $\gamma$ rays to light. This characteristic is desirable in so many fields. Over the years there has been increased interest in them especially in fast time measurement in nuclear physics^1^, for precision calorimetry in high-energy physics^2^ and for positron emission tomography in medical physics^3^. The interest has resulted in intense efforts channelled towards discovery, research and development of inorganic scintillator materials^1,4^. To be considered efficient, a scintillator is required to be stable upon exposure to radiation, posses high light yield, fast response, and high efficiency in absorbing radiation. The scintillation properties are closely linked to the structure of the material used. Moreover, the scintillation efficiency is controlled by the presence of defect and crystallographic properties, isotropic propagation of light in scintillation crystals notwithstanding^2^. Similarly, the scintillation yield, transport and luminescence yield are all dependent on the crystal structure of the scintillator^3^. Specifically, the energy transfer in the scintillator is a structure sensitive phenomena governed by carrier capture in deep and shallow traps, as well as other radiation-dependent defects^5^. Defects serve as trap for electrons and holes, interrupting energy transfer in the process. Indeed, previous investigations confirmed the existence of traps in scintillators, although a complete understanding of the energetics of these defects in most materials is still scarce. In view of the strong performance-structure relationship, it is important to understand the defect chemistry of the material, especially those that can be induced when the material is in operation^6,7^. This is required to improve existing and in design of resilient materials.

One of the most studied material used as scintillator is the Bismuth germanate $\hbox {Bi}_4\hbox {Ge}_3\hbox {O}_{{12}}$ (BGO)^8^. First synthesized in 1957, BGO is a cubic crystal with space group I43d possessing eulytine-type structure with $\hbox {BiO}_6$ octahedron and $\hbox {GeO}_4$ tetrahedron, an arrangement that can accommodate defects^1,9^. It has been extensively studied as a scintillator for various applications and found to emit light in the visible region upon exposure to ionizing radiation^10^. It is also known to possesses high density (7.13 g/$\hbox {cm}^3$) and high light yield (9000 photons/MeV), in addition to impeccable characteristics such as a short radiation length and absence of hygroscopicity^11^. These characteristics have resulted in the material finding application in tomography scanners and high-precision calorimeters used in the detection of electromagnetic radiation^12^. This advantages notwithstanding, its wide spread application in fields such as high energy physics is limited due to low response time (about 300 ns). Another issue is the presence of germanium in the system, which raises question about its cost^11^. Most of the issues encountered in this material is related to its crystal structure^13^. The arrangement of atoms in BGO is such that a number of charge-trapping sites exist. An understanding of the defect behaviour and formation in the material is required to enhance its performance and minimize deficiencies. Previously, thermo-luminescence experiments have been employed to characterize intrinsic defects in BGO. It was reported that the relative intensities of the glow peaks observed above room temperature depends on radiation dose and the presence of impurities. Certain defect types are suggested as trapping sites with further analysis revealing a range of trapping levels in pristine and doped BGO^14^. Atomistic simulations employing empirical pair-potential was used to calculate the formation energy of basic defects in BGO. Obtained results supports experiment observation of charge trapping defects in BGO^15^.

Theoretical methods been have applied successfully to describe various material characteristics related to defect and to calculate defect energetics of materials^13,16^. Specifically, density functional theory has proved to useful in determining band gaps and defect properties of scintillators^17^. Studies of this nature consider deviation from stoichiometry resulting from formation of intrinsic defects, whose presence determines the stability of the material in operation. The incorporation of dopant ions into perovskite and similar structures in a wide range of concentrations has been reported to improve properties and applicability of materials^18,19^. Specifically, the introduction of rare-earth elements (REE) has received immense attention due to its ability to modify electronic properties and luminescence in scintillator materials^20^. Moreover, the doping of BGO attracted attention due to the ability of its photons to interact with the material effectively and combine to form new photons with doubled energy and frequency^21^. Indeed, REE are interesting dopants for enhancing the properties of BGO. Among REE ions, $\hbox {Pr}^{3+}$, $\hbox {Nd}^{3+}$, $\hbox {Tm}^{3+}$ and $\hbox {Ce}^{3+}$ have received attention as a result of offering remarkable activator ion for luminescence^12,19^. Jazmati and coworkers^22^ investigated BGO: Ce samples implanted at linear no-threshold model at 77 K with He ions for manufacturing waveguides. They observed a phase change in the BGO, modifying its cubic structure to an anisotropic guide layer generated from the ’stress’ of the He beam deployment and, at the same time modifying the optical activity. Besides, Nd doped BGO demonstrates the properties that allow its use in the construction of solid-state lasers^23^. The advantages reported for these resultant materials notwithstanding, their practical applicability has been hindered by lack of detailed information about their microstructure and the position of the dopant atom in the system. The choice of the REE dopant employed for our investigation is guided by experimental findings. Different REE dopants have been reported to improve scintillation performance^12,22^. For instance, it has been show that the radiation resistance of BGO crystal was improved by Eu doping leading to faster induced absorption recovery^24^. In the same vein, Ce doping has been found to lead to occurrence of thermo-luminescence (TSL) peaks around room temperature (RT)^25^. Similarly, Nd, Tm and Ce are attractive dopant as they have been found to posses emission lines due to 4f–4f transmission from visible to near-infrared wavelength, hence are known as luminescence centers^26^.

**Figure 1:**
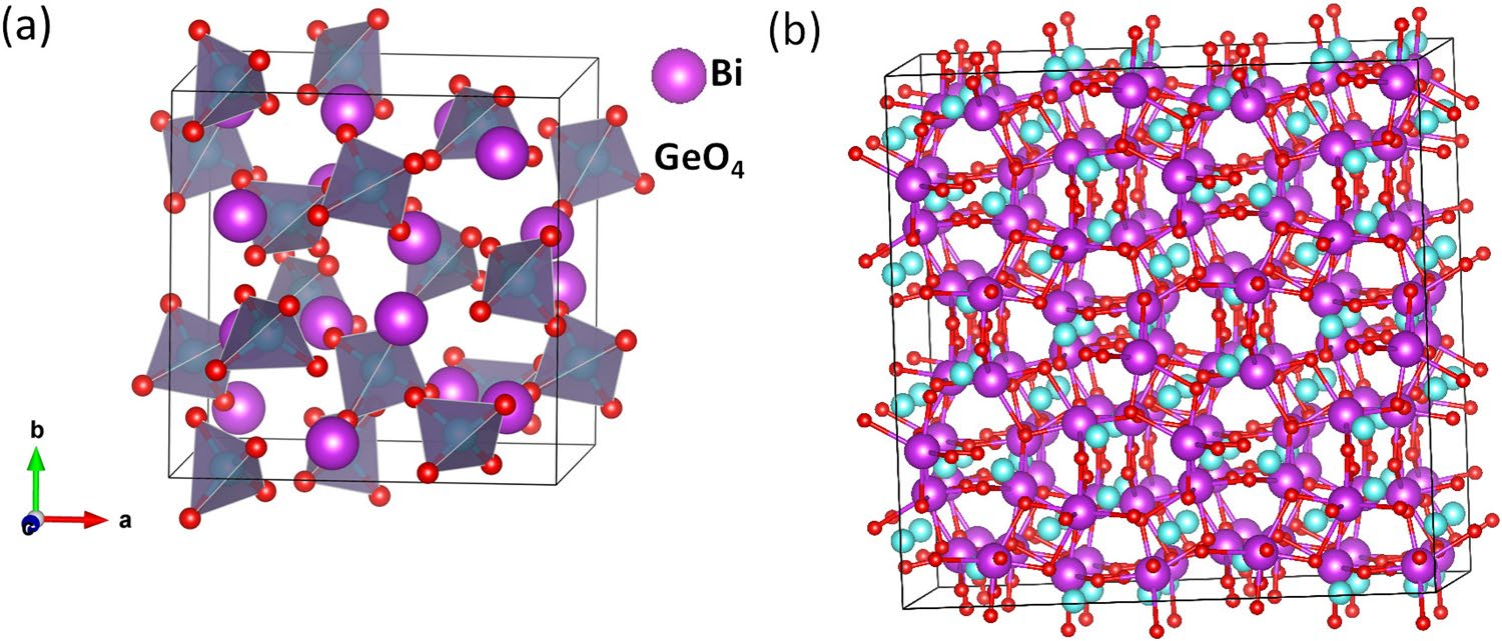
(**a**) Crystal structure of optimized BGO unit cell and (**b**) $2 \times 2\times$2 supercell used for defect simulations.

Previously, density functional theory (DFT)^13,27–29^ has been employed to study the electronic and optical properties of BGO. Also, attempts have been made to study the doping of BGO with rare earth elements in order to improve its applicability using DFT^12^. These investigations employed different flavours of the DFT within Generalized gradient approximation (GGA). It was reported that the major part of incoming energy is absorbed by oxygen (O) P electrons and transferred to bismuth (Bi) p states. All of these investigations reported band gaps that are below the experimental reported values. In order to accurately characterize the defect behaviour and optical properties of a system, it is important to obtain electronic properties that are close to experiment^30^. This work employs the hybrid PBE0 functional to study BGO. The hybrid functional, specifically the PBE0 has been used successfully to study systems where GGA has been found to underestimate band gaps^31^. In this work, we investigate the defect process in BGO and predict the stability of the different defect type, the effect of the prominent defect on factors affecting scintillation using density functional theory. Although this work focuses on BGO, inferences drawn from this investigation will aid understanding other materials used for similar applications. Moreover, it is expected that understanding of these kind of defects will help to optimize the efficiency of scintillators.

## Results and discussion

### Defect-free BGO

**Figure 2:**
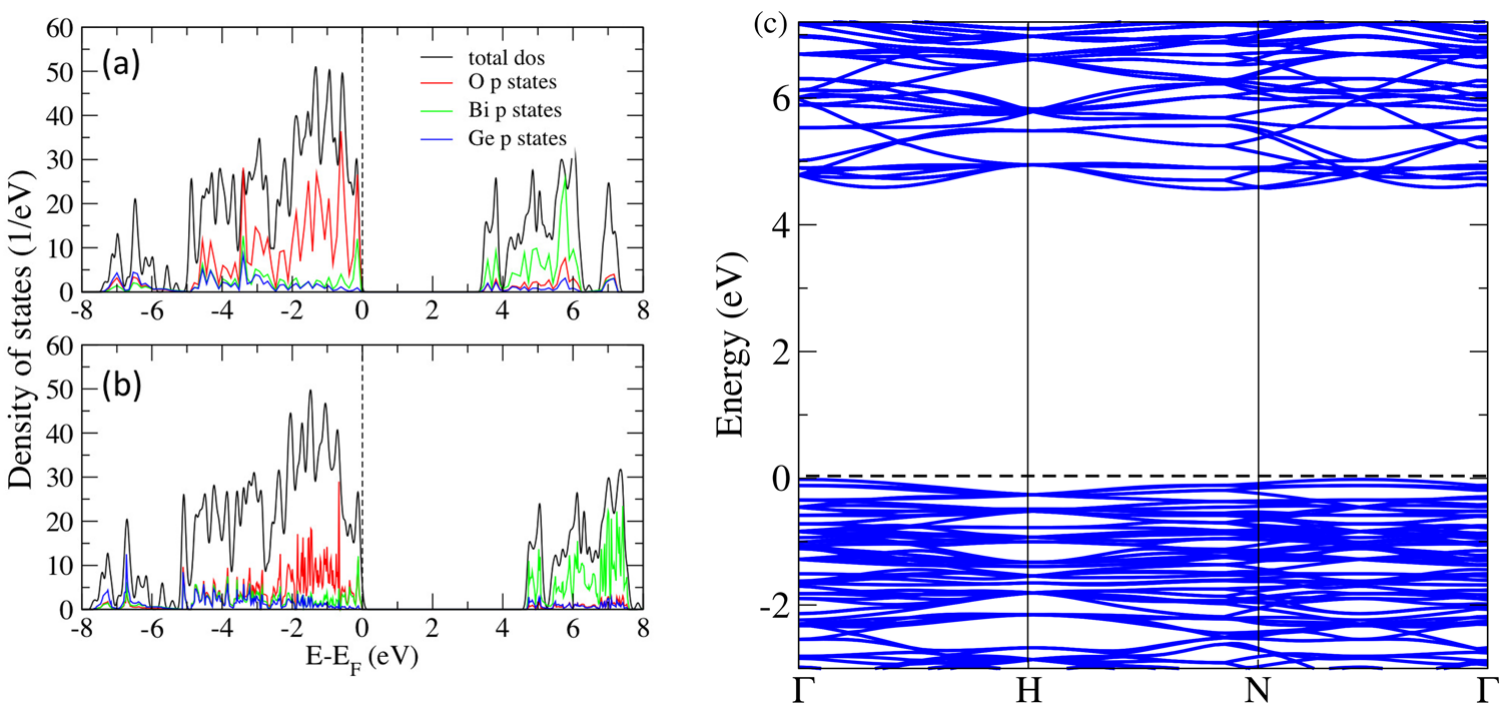
Total and partial density of states BGO calculated using (**a**) DFT-GGA (**b**) DFT-PBE0 and (**c**) band structure of pristine BGO calculated with DFT-PBE0.

The BGO system crystallizes in the cubic symmetry, comprising of a regular arrangement of $\hbox {GeO}_4$ tetrahedra sharing vertices with distorted $\hbox {BiO}_6$ octahedra, see Fig. 1a. The primitive unit cell contains two formula unit of 38 atoms^13^. Rodriguez and coworkers^28^ reported the crystal structure of BGO with experimental lattice parameter a = b = c = 10.513 Å. We begin our investigation by calculating the lattice parameter of pristine BGO and obtained a lattice parameter of 10.6 Å which is in good agreement with values reported from previous theoretical work^12^ and experimentally^28,32^. The BGO structure is such that two different Bi–O bonds exist due to the distortion of the the $\hbox {BiO}_6$ octahedron. The bond lengths for the pristine system are presented in Table 1. Observation reveals an agreement with values reported by experiment^32^. After validating our model for the calculation of the structural properties of defect-free BGO, we proceeded to calculate the electronic structure of the pristine system. The density of states in Fig. 2 offers insight into the chemical bonding in pristine BGO. Figure 2a presents the GGA calculated density of states, where we obtained a band gap of 3.4 eV comprising of a O-p states dominated valence band maximum (VBM) and a conduction band that comprises of hybridization of Bi-p and O-p states. Note that the calculated band gap is smaller than the experimental band gap of 4.58–4.7 eV^33,34^. Previous DFT investigations using GGA^12,28^ reported a band gap of 3.5 eV. Similar calculations using full-potential linearized augmented plane wave, taking into account spin-orbit interaction yielded similar band gap^13^. All previous work were unable to reproduce the experimental band gap. Correct prediction of the electronic properties especially fundamental properties of the gap, its width and states is important in determining the defect energetics^30^, optical properties and characterization of scintillation process^13^. In view of this, there is a need to consider another functional in a bid to improve the result.The DFT-PBE0 method with 12.5% exchange was employed and the obtained band gap of 4.6 eV is in better agreement with experiment than the one obtained with DFT-PBE. Figure 2b presents PBE0 calculated DOS for pristine BGO. The same features obtained for DFT-PBE are reproduced here with the only difference resulting from increase in the size of the band gap.The band structures (Fig. 2c) calculated along calculated along $\Gamma$ $\rightarrow$ H $\rightarrow$N $\rightarrow$ $\Gamma$ $\rightarrow$P $\rightarrow$ H|P high symmetry path of the Brillouin zone further supports the information obtained from the density of state plot shown in Fig. 2b. It is worth noting that our calculated band gap using the PBE0 significantly improves the band gap previously obtained using PBE-GGA functionals (see Table 1).

The electronic band gap of a material is a valuable feature that provides a deep understanding of its electronic, defect and optical properties. From our investigation, it was found that, the PBE0 approximation improves greatly the value of the band gap energy. Infact, PBE0 approximation improves, significantly, the calculated gap value better than the conventional GGA approximation. Llalic and coworkers^13^ have previously carried out first-principles calculations, including spin orbit coupling on BGO, while certain features were found to be improved in comparison to standard DFT, the band gap was underestimated. Using PBE0, key features of the electronic states are reproduced while a much improved band gap is also obtained. An accurate prediction of the band gap is important for the meaningful characterization of defect energetics^35,36^. Moreover, several DFT calculations^36–38^ on Bi-containing oxides have been successfully carried out, including investigations involving defect without the employing spin-orbit coupling.

**Table 1:** Calculated lattice parameters and the band gaps of pristine BGO Calculated equilibrium interatomic distances (Å) in BGO, compared to the experimental data. In parenthesis is the PBE0 calculated band gap.

	Our work	Exp^32^	Previous theory^28^
Bi-O	2.15 Å	2.14 Å	2.20 Å
	2.63 Å	2.61 Å	2.62 Å
Ge-O	1.75 Å	1.74 Å	1.76 Å
Band gap	3.4 eV	4.7 eV	3.5
	4.6 eV		

**Table 2:** Formation energies of intrinsic defect.

Defect type	Formation energy (eV)
O vacancy	2.30 (2.32)
Ge vacancy	7.33
Bi vacancy	3.61
O interstitial	3.26 (3.28)
Bi interstitial	5.70

**Figure 3:**
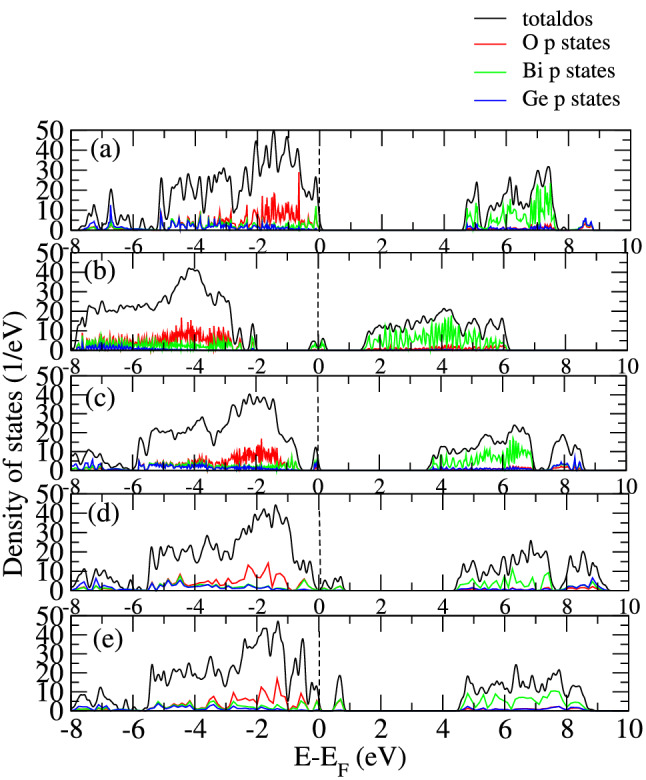
Total and partial density of states of (**a**) pristine BGO and BGO (**b**) antisite defect (**c**) O vacancy defect (**d**) Bi vacancy defect (**e**) Ge vacancy defect.

### Defect formation in BGO

Several important optical and luminescent characteristics are influenced by energy levels formed within band width of scintillators^31^. These levels are due to activator ions, impurities and point defects. Defects in materials are important as they control the physical, thermodynamic and electronic properties^31,39^. Intrinsic defects are disorders that can be thermally generated and not from doping or reaction with the environment^31^. They have been studied previously in similar systems and for similar applications^31,40^. For this investigation, we considered only isolated single defects for simplicity and excluded defect clustering. Similar approach was adopted in studies of defect for similar applications^40,41^.

The intrinsic defects in BGO include Bi, O, and Ge vacancy/interstitial. We modelled intrinsic defects in the BGO system by adding/removing an atom (of Bi, Ge, or O) to/from a $2 \times 2\times$2 supercell (Fig. 1b). The formation energy of the different intrinsic defects considered are calculated thus:

1 $$\begin{aligned} E= E_{defective}- E_{Pristine} \pm \mu \end{aligned}$$

$E_{defective}$ and $E_{Pristine}$ are the total energies of defective and defect-free supercells, respectively. $\mu$ is the chemical potential of the atoms considered. The sign in equation is $+$ for vacancy and − for interstitial. A combination of one vacancy and one interstitial defect makes a Frenkel pair. To calculate, the Frenkel energy, we first investigate formation energy of the different possible O vacancy sites and find the most favourable site, considering O vacancies in the $\hbox {BiO}_6$ and $\hbox {GeO}_4$ coordination. Generally, we find that the O vacancy formation energies are slightly lower for Bi coordinated O atoms than Ge coordinated ones. We obtained O vacancy formation energy of 2.32 and 2.30 eV for Ge and Bi coordinated O atoms respectively. The same approach used in determination of stable site for O vacancy formation is adopted in finding the most favorable interstitial site. The O interstitial formation energy is calculated to vary between 1.47 and 1.61 eV for the possible scenarios considered. From the most energetically favorable configurations for the O vacancy and interstitial, we obtained the O Frenkel energy for BGO to be between 0.83 eV (2.30–1.47 eV) and 0.71 eV ( 2.32–1.61 eV) respectively for the Ge and Bi sublattices. From the calculation of the O defect formation energies, we find that the $\hbox {GeO}_4$ sublattice is stable, hence most defects are associated with the $\hbox {BiO}_6$ sublattice. In Table 2, we list the calculated formation energies of O, Ge and Bi vacancies and interstitials.

Another type of defect considered is the antisite defect. Antisite defects are a common defect type in many oxide based compounds and have been reported experimentally and theoretically^35,42^. To generate the antisite defect, we placed Ge in a site that is originally occupied by Bi and vice versa. Similar approach has been adopted in study of antisite defects in oxides^35,43^. The antisite defect formation energy $\hbox {E}_{antisite_formation}$ is defined as the energy difference between energy of configuration with an antisite defect ($\hbox {E}_{{antisite}}$) and the energy of the pristine system^43^.

2 $$\begin{aligned} E_{antisite_formation}= E_{antisite}- E_{Pristine} \end{aligned}$$

We obtained the energy required to form the $\hbox {Bi}_{{Ge}}$ and $\hbox {Ge}_{{Bi}}$ antisites as − 0.48 eV and 4.30 eV respectively. A comparison of the Frenkel and antisite defect energetics shows that the $\hbox {Bi}_Ge$ antisite defect are much easier to form than the O Frenkel. This observation suggests that the $\hbox {Bi}^{3+}$ is likely to be charge trap site and the main optically active constituent of the BGO.

The electronic structure of scintillators is a crucial factor in the luminescence properties of these components. In order to achieve the best possible efficiency, the band gap needs to be narrowed^13^. To investigate the origin of the electronic structure modification due to intrinsic defects, we analyse the density of states as shown in Fig. 3 and compare with some of the prominent defects. As mentioned earlier, our calculated band gap for pristine BGO is 4.6 eV (shown again in Fig. 3a). Our analysis of the electronic structure was carried out on the most favourable defect types. Here we consider the different vacancies and antisite defect. The presence of vacancy introduces defects states within the band. Figure 3b–e shows the densities of state for the BGO with antisite defect, O vacancy, Bi vacancy and Ge vacancy respectively. The plots show that the position of the defect state induced depends on the type of defect. Generally, the overall shape of the DOS and composition of the bands are unaltered for the vacancy defects. However, for the case of system with antisite defect, there is a downward shift in the conduction band into the band gap when compared with the pristine case. Similar downward shift was observed for the valence band culminating in a split into discrete bands. The states introduced are mainly as a result of Bi p states. This suggests that charge carrier thermalization can be suppressed thereby leading to intra band luminescence of carriers.

**Figure 4:**
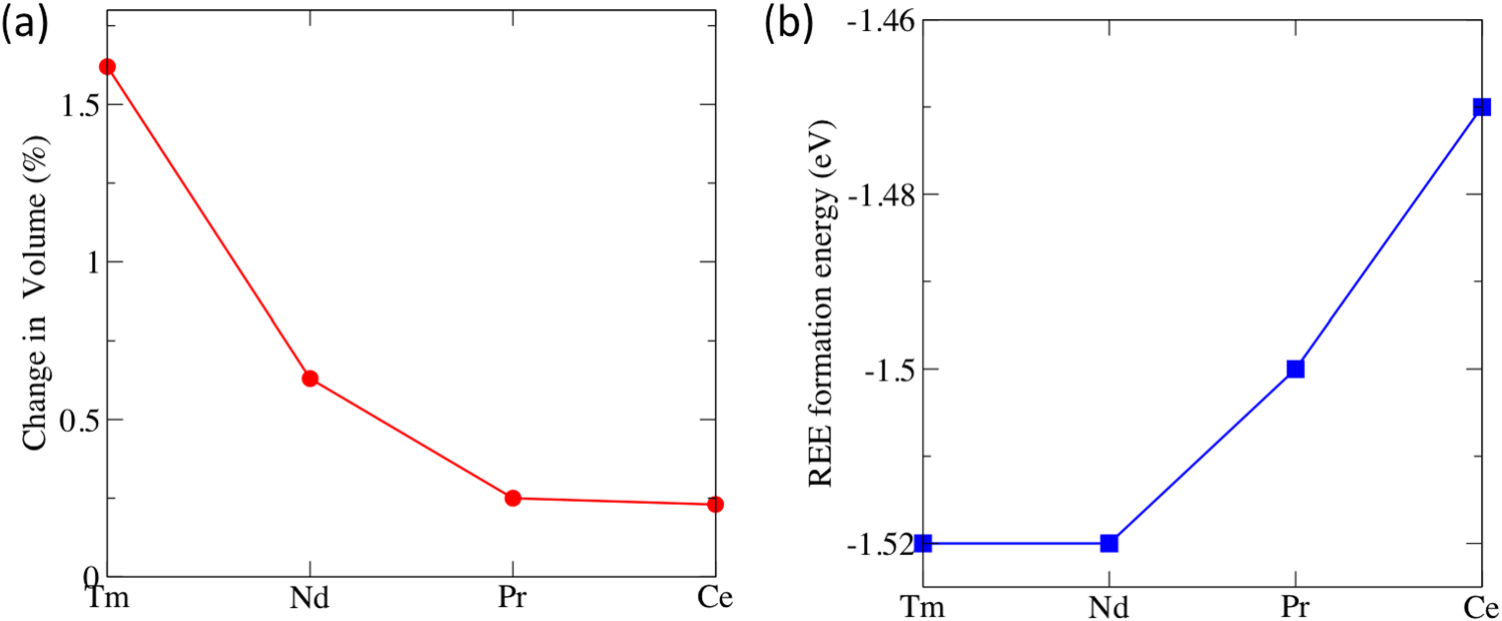
(**a**) Change in volume of BGO upon doping with REE (**b**) Formation energy of doped REE systems.

### Cation doping

The trivalent Bi cation has a suitable ionic size to accommodate the REE ion and has been found to influence properties of scintillators^44^. This makes them suitable dopants for the BGO system^22^. To substitute the REE atom at the Bi site, we considered all the non-equivalent Bi sites and proceeded with our calculation with the the most stable site for doping. For our investigation, we considered REE = Ce, Pr, Nd, and Tm as dopant elements;all considered in previous experimental studies^22,24^. The REE atoms are considered to replace Bi atom culminating in a REE/Bi ratio of 1/64 in the supercell. For the considered dopants, the size of the ionic radii decreases from Ce (1.01 Å) to Tm (0.88 Å) resulting in a decrease in REE-O bond length. Overall, the doping of BGO with REE affects the structural properties of BGO minimally. Figure 4a shows the percentage change in volume of relaxed structure of the BGO:REE. We find that the volume is not hugely changed upon doping with REE, this is because the ionic radii of the Bi and the REE ions are similar and the concentration of REE introduced is small. Our simulation revealed a maximum change of 1.62% (Tm). The minimal change in volume is found for BGO:Ce. This is expected as the ionic radii of Ce (1.01Å) is similar to that of Bi (1.03Å)^45^. Overall, the volume of the system decreases as the ionic radii of REE decreases (REE doping leads to reduction in volume). Our investigation proceeded with the determination of the stability of the dopant systems by evaluating the formation energy of the doping with REE. We calculated the formation energies for the different dopant systems using^16^

3 $$\begin{aligned} E_{formation}= E_{REE} - E_{pristine} + \mu _{Bi} - \mu _{REE} \end{aligned}$$

where $\hbox {E}_{{formation}}$, $\hbox {E}_{{REE}}$, $\hbox {E}_{{pristine}}$ are the formation energy of substitution of REE, energy of system with REE dopant and energy of pristine system respectively. $\mu _{Bi}$ and$\mu _{REE}$ are the chemical potential of the Bi, REE and oxygen atoms respectively. The value calculated quantifies the ease of substitution of the dopant element in the BGO system. Figure 4b shows the calculated dopant substitution formation energy as a function of the different dopant elements. We observed that the value of $\hbox {E}_{{formation}}$ gradually reduces as the size of the REE substituted. However, the Nd doped system does not follow this trend. To shed light into the reason for this deviation, the REE-O bond length is examined. We find that although the REE–O length increases as the ionic radii^45^ of REE increases, Nd–O bond length is similar to Tm–O bond length. Jazmati et al.^22^ studied the role of rare earth on properties of BGO and found unique properties in Nd doped BGO. It was found to modify its cubic structure of BGO to an anisotropic guide layer thereby exhibiting non linear optical behaviour.

**Figure 5:**
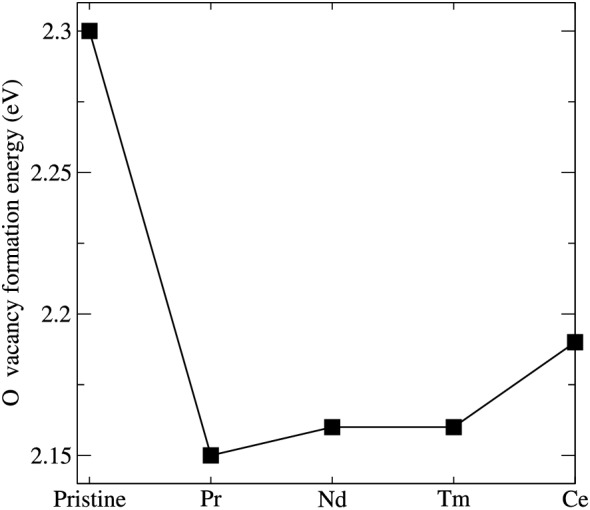
O vacancy formation energy of pristine and REE doped BGO.

**Figure 6:**
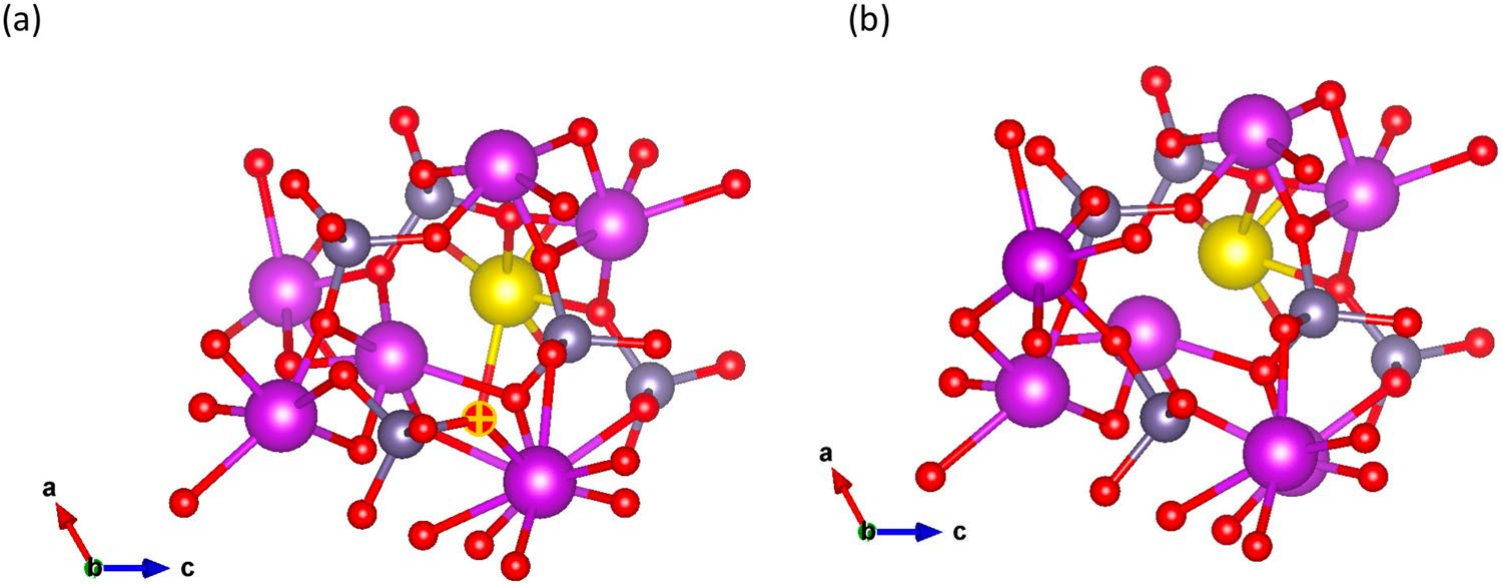
Arrangement of atom around REE atom (**a**) before O vacancy (**b**) after O vacancy. The yellow ball depicts Pr atom and marked O is the atom removed.

The existence of oxygen vacancy in BGO is inherent and its formation in pristine BGO has been discussed earlier. Moreover, the local distortion caused by the REE dopant (as a result of difference in size) may affect the stability of surrounding O atoms^16^. The effect of location distortion induced as result of REE doping has been reported in oxides^16,46^. Hence it is important to quantify the stability of the O vacancy in the system. Upon substitution of the REE dopant, the O vacancy formation energy is calculated using Eq. (5)

4 $$\begin{aligned} E_{V^1_o}= E_{REE} - E_{(REE+Vac)}- \frac{1}{2}\mu _{O} \end{aligned}$$

where $\hbox {E}_{V^1_o}$, $\hbox {E}_{{REE}}$ and $\hbox {E}_{(REE+Vac)}$ are the energy of formation of O vacancy in a system doped with REE, energy of system with REE dopant at the Bi site only and energy of system with REE dopant with O vacancy, respectively and $\mu _{O}$ is the chemical potential of O. The O vacancy formation energy as a function of dopant is presented in Fig. 5. We considered O sites close to the REE dopant (see Fig. 6). Our investigation shows that all of the considered dopant systems are less stable in the presence of O vacancy than the pristine BGO system with the Ce-doped system being the most stable of all the considered REE dopants. It is worth noting that while the O vacancy formation energies obtained for Tm, Nd and Ce follow a trend that mimicks the ionic radii of the dopant (see Fig. 4a), Pr does not follow this trend. O vacancy formation energy is formed more easily in Pr doped system compared to the other dopants. Overall, the REE with largest ionic radii posses the highest O vacancy formation energy, see Fig. 5. The very small difference in O vacancy formation energy in the dopant system suggests similar behavior in the system, and shows that even with low concentration of dopant, the stability of the BGO system can be altered in the presence of defect like O vacancy.

### Optical properties

**Figure 7:**
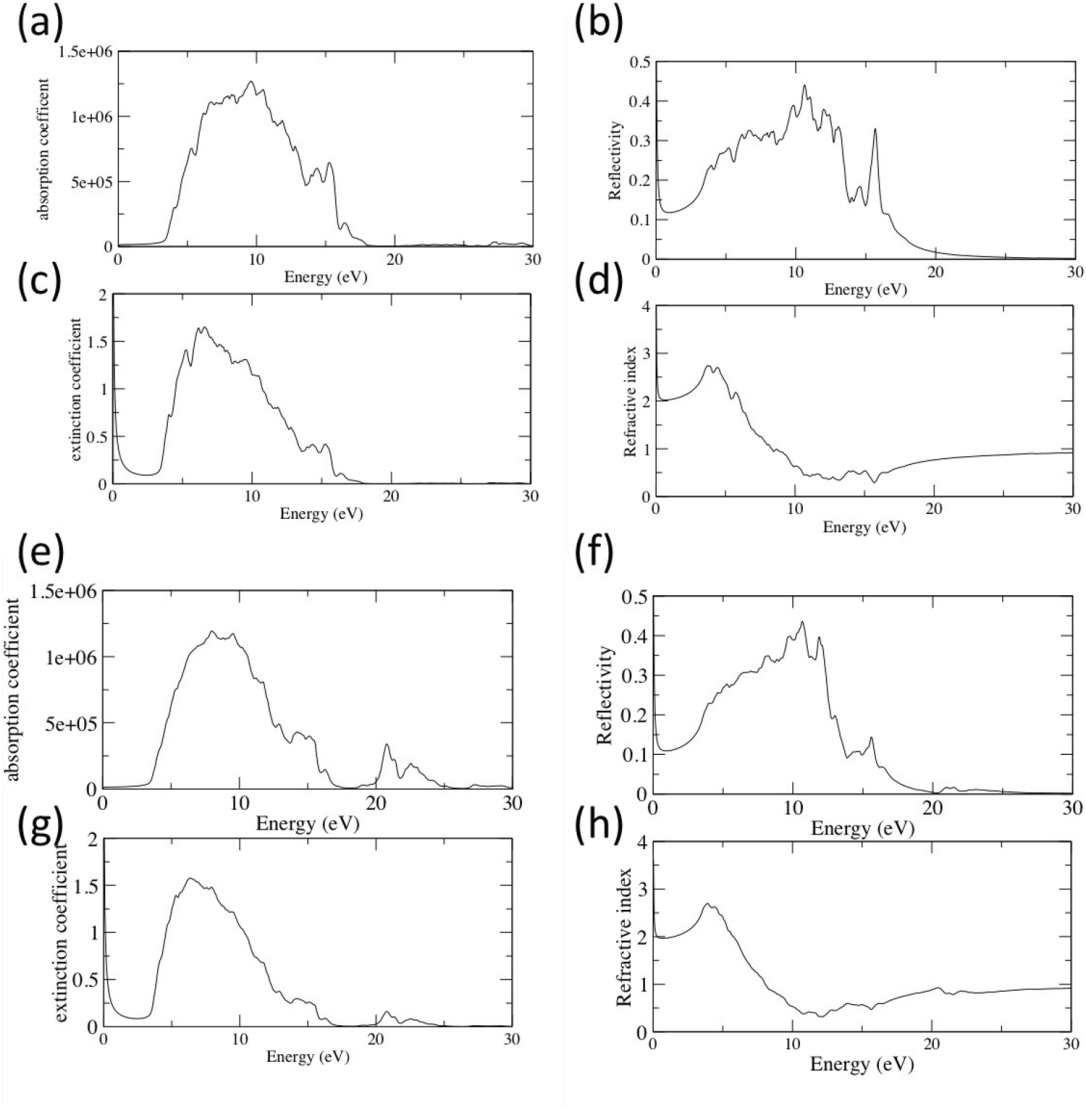
The calculated of (**a**) absorption coefficient (**b**) reflectivity, (**c**) extinction coefficient and (**d**) refractive index of pristine BGO. The lower panel presents (**d**) absorption coefficient (**e**) reflectivity, (**f**) extinction coefficient and (**g**) refractive index of Ce-doped BGO.

In this section, we discuss the optical properties of BGO within the photon energy 0–30 eV, calculated using the PBE0 functional. Here, we calculate the absorption coefficient, reflectivity, extinction coefficient and refractive index. The ability of a material to store electrical charges is often quantified by its dielectric constant ($\epsilon$). It is a significant optical property of a material. It comprises of real and imaginary part as described above. The imaginary part is directly proportional to absorption spectrum. The absorption spectra is shown Fig 7a. Analysis of the absorption spectra characterized the highest intensity to be within 4–13 eV with the peak at 8 eV. Above 13 eV, we notice a medium intensity with a smaller peak at 15.5 eV. Above 20 eV there is negligible intensity. Where there is zero absorption intensity, it implies that at the corresponding energy there is absence of dispersion resulting in maximum absorption. The nature of reflectance of incident radiation on the BGO is described by its reflectivity. The reflective spectra is shown in Fig. 7b. Similar to the absorption spectra, it is characterized by three regimes with the peak attaining maximum at about 12 eV. The obtained spectrum reproduced features observed experimentally^47^. Not only is the shape of the spectrum similar, the peaks are situated at comparable energies. The extinction properties are shown in Fig. 7c, it elucidates the absorption losses at particular ranges of incident electromagnetic spectrum. From our calculated spectrum, it can be seen that the extinction coefficient increases gradually and is maximum at 6.4 eV from which its value declines rapidly further. We obtained a refractive index of 2.6 at 3.5 eV, see Fig. 7d. This value is in agreement refractive index obtained in experiment^47^.

We present the absorption, reflective, extinction and refractive spectra of the most stable dopant system (Ce-doped BGO) in Fig. 7e–h. A comparison of the spectra with those obtained for pristine BGO shows some similarities. For example, the peaks for the four spectra are of the same order of magnitude and obtained at almost the same energy. The similarities notwithstanding, we observed contrast in certain features. For example, while the absportion spectra has no intensity above 20 eV for pristine BGO, we observed intensity upto 25 eV for Ce-doped BGO. Similar observation is made for reflectivity. The fact that upon doping the BGO system with very low concentration of Pr we observe contributions to the optical adsorption suggests that the low light yield and slow response which has plagued the applicability of the BGO crystals as scintillators for positron electron tomograhy can be improved by defect engineering as well as improved crystal growth.

## Conclusion

We have investigated the energetics of the formation and electronic properties of isolated defects in BGO using first-principles method. The investigation is essential to identify routes to improve scintillator performance. The calculation employed the hybrid PBE0 functional with 12.5% Hatree Fock exchange component to reproduce experimental band gap since PBE functional has been found to underestimate this very important property. $\hbox {B}_{{Ge}}$ antisite defect was predicted to be the most favorable defect of all considered defect types and introduces states within the band gap, introduces states within the valence band while shifting the bands relative to the states obtained for the pristine BGO, confirming the presence of trap sites in BGO. Furthermore, our investigation examined different rare earth dopants and their formation energies. We found O vacancy formation to be easily formed in REE doped system than in the pristine system. We also investigated the optical properties using approach that best reproduce the band gap of pristine BGO and noted variations in different regions of the photon energy spectra. Our investigation shed light on the role of defects and how using cation doping can be a route to control the stability of BGO.

## Methods

All calculations were carried out using the ab initio density functional theory using the projector augmented wave method of the Vienna Ab initio Simulation Package^48^. A plane wave basis set with a cut-off energy of 520 eV and a $4 \times 4\times$4 Monkhorst-Pack generated sets of k-points were used for optimizing $2 \times 2\times$2 supercell of BGO. We tested the convergence of the cutoff energy and K-points and found that increasing both produced negligible difference in the calculated results. (see Figs. S1 and S2 in Supplementary information. Both the lattice parameter and atomic positions relaxed with the energies and the forces of each ion were converged within $1.0 \times 10^{-5}$ eV/atom and 0.01 eV/Å, respectively. In order to reproduce the electronic properties as reported by previous experiment, hybrid functional, PBE0 (a scheme that combines a fraction of Hartree-Fock exchange with PBE exchange) calculations was carried out after Perdew-Burke-Ernzerhof (PBE)^49,50^ was unable to correctly predict the band gap of BGO. Defects are simulated by removing an O atom from a $2 \times 2\times$2 supercell constructed from the cubic structure. The size of cell used for the simulation of O vacancy is large enough to prevent defect-defect interactions.A minimal deviation in formation energy (about 1 meV) was found when a larger $3 \times 3\times$3 supercell was used.

Furthermore, we calculated the optical properties within the random phase approximation (RPA) by evaluating the complex dielectric tensor $\epsilon$. The dielectric tensor comprises of real and imaginary part, with the imaginary part being directly proportional to the optical spectrum of the system. Also, the dielectric function can be expressed in terms of refractive index n, extinction coefficient k as follows^13^:

5 $$\begin{aligned} \varepsilon = \varepsilon _{1} + i\varepsilon _{2} = (n+ ik)^2 \end{aligned}$$

Knowledge of the energy dependence of this component will help to quantify the response of the material to incident energy and the subsequent decay of that light propagating through the absorbing medium^51^.

## Supplementary Information

Supplementary Information.

## Data Availability

The datasets used and/or analyzed during the current study available from the corresponding author on reasonable request.
