# Supplementary material for: First-principles studies of defect behaviour in bismuth germanate
Source: Sci Rep. 2022 Sep 21;12:15728. doi: 10.1038/s41598-022-18586-x (PMC9492720; doi:10.1038/s41598-022-18586-x)
Supplement: Supplementary file 1 — Supplementary Information. [file 41598_2022_18586_MOESM1_ESM.docx]

**Supplementary information for first-principles studies of defect behaviour in bismuth germanate**

**Salawu Omotayo Akande**1,* **and Othmane Bouhali**2

Figure S1: Total energy vs Encut plot

Figure S2: Total energy vs Kpoints
